# Insect fauna including unrecorded species in Ulleungdo, South Korea

**DOI:** 10.3897/BDJ.11.e100783

**Published:** 2023-05-11

**Authors:** Min Hyeok Won, Jae Won Choi, WooJun Bang, DongYeol Lee, Min Ki Moon, Young-Kun Kim, Donguk Kim, Dooyoung Kim, Sang Jae Suh, Kwang Shik Choi

**Affiliations:** 1 Research Institute for Dok-do and Ulleung-do Island, Kyungpook National University, Daegu, South Korea Research Institute for Dok-do and Ulleung-do Island, Kyungpook National University Daegu South Korea; 2 School of Life Science, BK21 FOUR KNU Creative BioResearch Group, Kyungpook National University, Daegu, South Korea School of Life Science, BK21 FOUR KNU Creative BioResearch Group, Kyungpook National University Daegu South Korea; 3 School of Biological Sciences, Seoul National University, Seoul, South Korea School of Biological Sciences, Seoul National University Seoul South Korea; 4 School of Applied Biosciences, Kyungpook National University, Daegu, South Korea School of Applied Biosciences, Kyungpook National University Daegu South Korea; 5 Institute of Plant Medicine, Kyungpook National University, Daegu, South Korea Institute of Plant Medicine, Kyungpook National University Daegu South Korea; 6 Research Institute for Phylogenomics and Evolution, Kyungpook National University, Daegu, South Korea Research Institute for Phylogenomics and Evolution, Kyungpook National University Daegu South Korea

**Keywords:** Insecta, island, diversity, database, new records, Palearctic

## Abstract

**Background:**

Ulleungdo harbours a unique ecosystem owing to its isolation from the mainland alongside its maritime climate. The island, formed via volcanic activity, is the largest island in the East Sea of Korea and retains a primeval forest. The ecosystems are being destroyed owing to increasing human activity on the island. Therefore, through the investigation of the insect fauna of Ulleungdo, we tried to provide information that can be the basis for understanding the island ecology of Ulleungdo. This survey was conducted four times between April and October in 2020 at Seonginbong.

**New information:**

The findings of the survey regarding insect fauna at Seonginbong, Ulleungdo included 10 orders, 105 families, 216 genera and 212 species, of which 12 families, two subfamilies, 13 genera and 74 species were previously unrecorded. The data have been registered in the Global Biodiversity Information Facility (GBIF; www.GBIF.org).

## Introduction

In general, island ecosystems are isolated and have limited resources, which consequently results in simpler food chains than those in inland ecosystems (Polis et al. 1997). Therefore, the interrelationship between predators and prey in island ecosystems can be compared more clearly than in inland where the relationship is complex (Simberloff 1974). In addition, the biota of island regions is expectedly distinct from inland biota as the former is affected by oceanic climates, unlike inland ecosystems, which are affected by continental climates. However, islands surrounded by oceans are vulnerable to the effects of climate change, such as rising sea levels due to global warming, changes in temperature and precipitation, increasing incidences of unpredictable tropical cyclones and El Nino (Ahn 2011). In case such climate change continues in Korea, boreal plants in temperate regions in the Northern Hemisphere are expected to become extinct, whereas the distribution of temperate plants is expected to expand rapidly (Kim et al. 2022). Furthermore, human activities, such as island development and the influx of foreign species have added to the threat of biodiversity reduction in island ecosystems (Veron et al. 2019). For this reason, island ecosystems require constant monitoring.

Ulleungdo and its subsidiary islands are the sole island areas located in the East Sea of Korea, thereby representing the only island ecosystem in the East Sea. Ulleungdo is located in the southwest of the East Sea (37°30'N, 130°52'E) and at a distance of 130 km from the Korean Peninsula. The island was formed via volcanic activity and is an ocean island that has never been connected to land. Seonginbong (984 m) is located at the centre of Ulleungdo. The primeval forest of Seonginbong is an ecologically stable climax forest and includes plants that are unique to Ulleungdo (Cho et al. 1993).

An investigation about the insect fauna of Ulleungdo was first conducted by Cho, who reported four families and 16 species of butterflies (Cho 1929). Subsequent studies have identified various taxa, including 95 families and 345 species (Kim 1971), 125 families and 574 species (Lee and Kwon 1981), 141 families and 691 species (Kwon et al. 1996), 154 families and 828 species (Lee and Jung 2001), 153 families and 841 species (Lee et al. 2006), 81 families and 242 species (Lim and Lee 2012) and 96 families and 433 species (Lim et al. 2013). According to Lim et al. (2013), a total of 18 orders, 179 families and 1,177 species of insects were recorded on Ulleungdo during the survey period from 1929 to 2013. In addition, a recent survey by the National Institute of Biological Resources (National Institute of Biological Resources 2021b) involving aquatic insects in Ulleungdo identified 32 species of aquatic insects, including Ephemeroptera, Trichoptera and Plecoptera. Given its distinct geography as an island region, Ulleungdo has poor accessibility. In addition, owing to severe weather disturbances, such as typhoons and high waves, periodic insect fauna surveys have been difficult to conduct. Although there have been several surveys in the past, the overall insect fauna survey has not been carried out since the study of Lim et al. (2013) and the most recent survey (National Institute of Biological Resources 2021b) was not a general survey of insect fauna, but the aquatic environment. Therefore, there is a need to update the insect fauna data and it is possible that there are still many unrecorded insect species on Ulleungdo. Herein, a comprehensive survey of the insect fauna inhabiting Seonginbong in Ulleungdo was conducted using different collection methods and a species list, including that of previously unrecorded species, was prepared.

## Sampling methods

### Study extent

**Figure 1.**
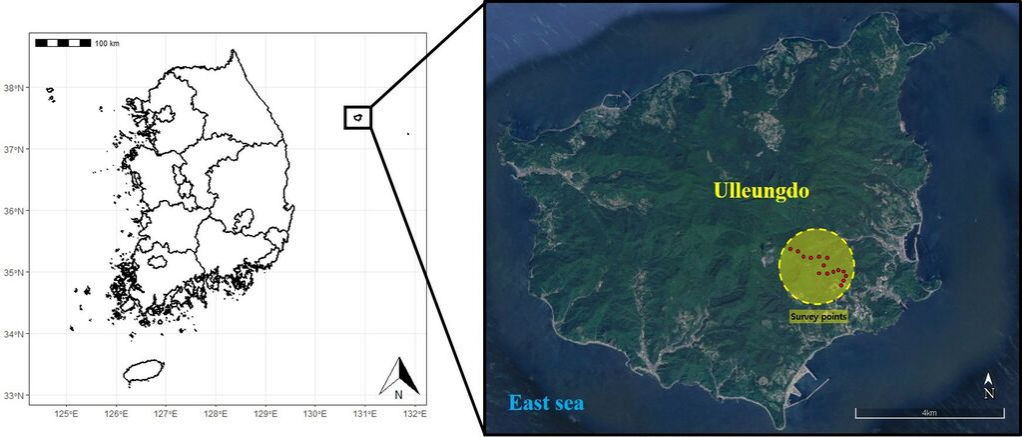
Location of Ulleungdo, Korea.

Throughout 2020 (April, July, August and October) four expeditions were carried out to collect data at Seonginbong (37°29'52.81''N, 130°52'03.72''E) in Ulleungdo (Fig. 1). Fifteen collection points were designated along the altitude of Seonginbong and four collection methods were used: light trap, molasses traps, pit-fall traps and sweeping.

### Sampling description

Light trap was conducted at collection point 1 (lowland), collection point 6 (midland) and collection point 15 (highland). After fixing a tripod (height: 1 m) inside a tent (height: 1.7 m), a 400 W high-voltage mercury lamp was connected with a tripod. Samples attracted by ultra-violet light from a mercury lamp were collected by a hand-collecting method. Light trap was operated after 20:00 h when the sun had completely set and it was operated for about 1 hour at each point.

Molasses traps were conducted at all 15 collection points. The distance between each collection point is about 50 m. Tissue soaked in attractant was put in a mesh net and hung on a tree. The attracted samples were collected by a hand-collecting method. Molasses, made by mixing sugar, glacial acetic acid and grape juice, was used as an attractant (Scheller 1984, Singh et al. 2013, Dar et al. 2020). Molasses traps were installed at 15 points by altitude and maintained for 24 hours. Insects attached to the traps were collected one day after traps installation.

Pit-fall traps were also conducted in all 15 collection points. A plastic cup (diameter: 9.2 cm; height: 13.5 cm; volume: about 475 cm^3^) containing an attractant was buried at the same level as the ground. Molasses, pork and octopus were used as attractant and, in the case of molasses, the same molasses as the molasses trap were used. Each attractant was separately put into a plastic cup and three types of pit-fall traps were installed at regular intervals of 3 m. Three pit-fall traps were installed for each attractant at one point and a total of 45 traps were installed at 15 points. Pit-fall traps, like molasses traps, installed at 15 points by altitude, were maintained for 24 hours from the time of installation and then the insects in the traps were collected next day.

Sweeping was conducted continuously while going up from collection site 1 to 15 and samples were collected by sweeping an insect net (pole: 2.5 m; net diameter: 50 cm; net length: 110 cm). Sweeps were performed at least 50 times for each point. The samples collected in the insect net were transferred into the conical tube using an insect aspirator. Sweeping was carried out while climbing the Seounginbong during the daytime.

Collected samples were stored in conical tubes containing 70% ethanol. Large insects, such as some Lepidoptera species, were stored in glassine paper and frozen to prevent damage. Afterwards, the collected samples were moved to the Animal Systematics & Taxonomy Laboratory at Kyungpook National University. The species were identified by referring to various references (Hardy and Takahashi 1960, Shin 2001, Park et al. 2012, An 2013, Cho 2015a, Cho 2015b, Cho 2015c, Jang et al. 2015, Baek 2016, Dong 2017, National Institute of Biological Resources 2021a). In order to confirm that the identified species are unrecorded species of Ulleungdo, they were checked through the references which including a list of insect species previously investigated on Ulleungdo (Kim 1971, Lee and Kwon 1981, Lee et al. 2006, Lim and Lee 2012, Lim et al. 2013).

**Database update**: A list of 212 insect species collected from Ulleungdo in 2020 was prepared and the data were registered in the Global Biodiversity Information Facility (GBIF).

## Geographic coverage

### Description

This survey was conducted at Seonginbong, Ulleungdo.

### Coordinates

37°29'10'' and 37°29'54''N Latitude; 130°52'03'' and 130°53'39''E Longitude.

## Taxonomic coverage

### Taxa included

**Table:** 

Rank	Scientific Name	Common Name
kingdom	Animalia	Animals
phylum	Arthropoda	Arthropods
class	Insecta	Insects
order	Blattodea	
order	Coleoptera	
order	Dermaptera	
order	Diptera	
order	Hemiptera	
order	Hymenoptera	
order	Lepidoptera	
order	Mantodea	
order	Orthoptera	
order	Trichoptera	

## Usage licence

### Usage licence

Creative Commons Public Domain Waiver (CC-Zero)

## Data resources

### Data package title

2020_Ulleungdo_insect_list

### Resource link

https://doi.org/10.15468/bvxcjq

### Number of data sets

1

### Data set 1.

#### Data set name

2020_Ulleungdo_insect_list

#### Data format

CSV.

#### Download URL

https://www.gbif.org/dataset/b2ccb272-cd23-4c1e-8c07-660ff0099fff

#### Description

The dataset (Kyungpook National University Animal Systematics & Taxonomy Laboratory 2023) included 10 orders, 105 families, 216 genera and 212 species of insects. This survey was prepared four times (28/04/2020-03/05/2020, 05/07/2020-08/07/2020, 28/08/2020-31/08/2020, 01/10/2020-04/10/2020) at Seonginbong of Ulleungdo. The collection methods used include sweeping, light trap, pit-fall trap and molasses trap.

**Data set 1.** 

Column label	Column description
taxonID	An identifier for the set of taxon information (data associated with the Taxon class).
scientificName	Full scientific name.
taxonRank	The taxonomic rank of the most specific name in the scientificName.
kingdom	The full scientific name of the kingdom in which the taxon is classified.
phylum	The full scientific name of the phylum or division in which the taxon is classified.
class	The full scientific name of the class in which the taxon is classified.
order	The full scientific name of the order in which the taxon is classified.
family	The full scientific name of the family in which the taxon is classified.
genus	The full scientific name of the genus in which the taxon is classified.
specificEpithet	The name of the first or species epithet of the scientificName.
infraspecificEpithet	The name of the lowest or terminal infraspecific epithet of the scientificName, excluding any rank designation.
vernacularName	Common or vernacular name in Korea.
occurrenceID	Unique identifier of the occurrence.
basisOfRecord	State of the recorded specimen.
countryCode	Country code.
stateProvince	Province in which the specimen was collected.
county	County in which the specimen was collected.
locality	Locality in which the specimen was collected.
decimalLatitude	Geographic latitude of the collection site.
decimalLongitude	Geographic longitude of the collection site.
geodeticDatum	The ellipsoid, geodetic datum or spatial reference system (SRS) upon which the geographic coordinates given in decimalLatitude and decimalLongitude are based.
coordinateUncertaintyInMetres	The horizontal distance (in metres) from the given decimalLatitude and decimalLongitude describing the smallest circle containing the whole of the Location.
eventDate	Date of sampling period.
identifiedBy	Identifier for the specimen.
recordedBy	A list (concatenated and separated) of names of people, groups or organisations responsible for recording the original Occurrence.
identificationRemarks	Comments or notes about the Identification.

## Additional information

### Results and Discussion

**Figure 2.**
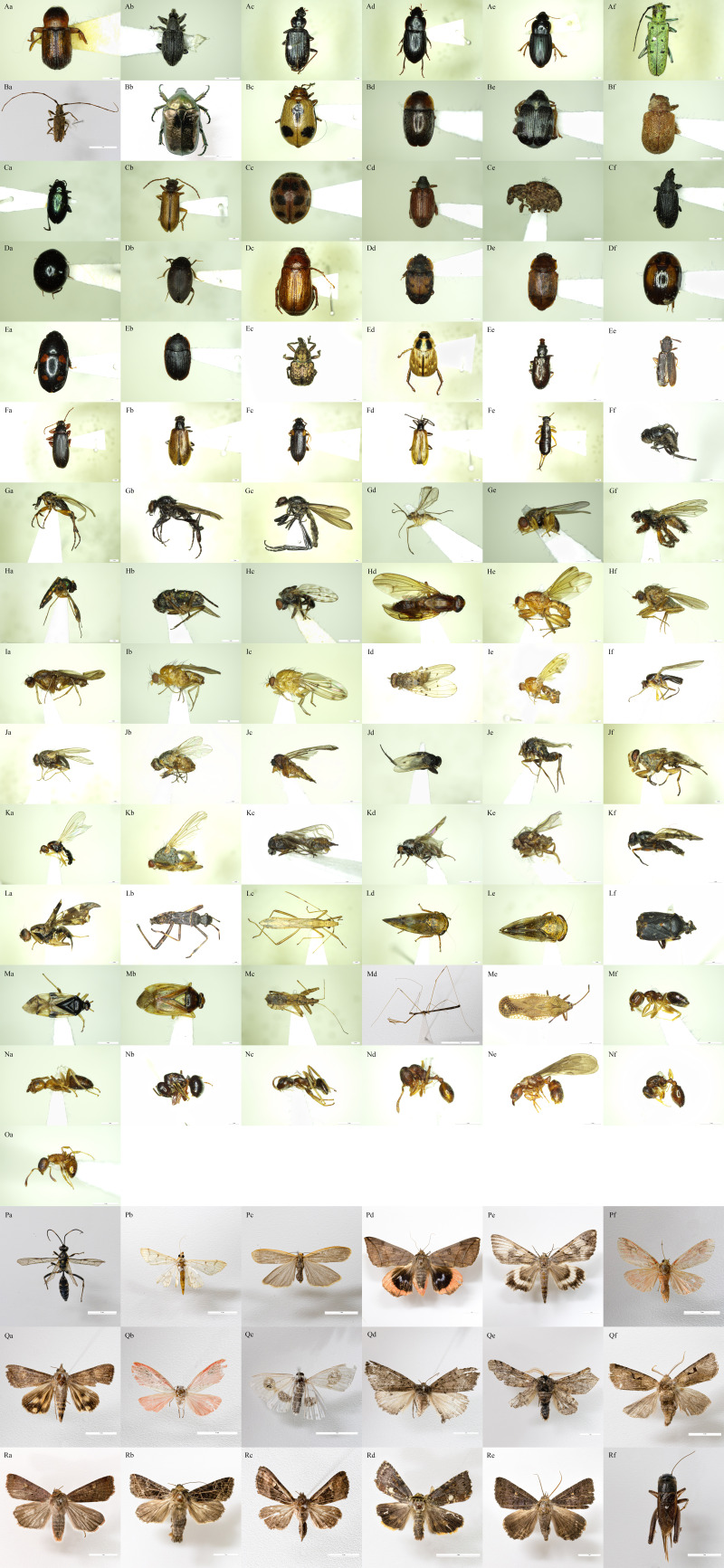
Newly-recorded insect species from Seonginbong, Ulleungdo in 2020. **Aa.** *Bostrichidae* sp.; **Ab.** *Apioninae* sp.; **Ac.** Colpodes (Gyrochaetostylus) atricomes; **Ad.** *Nipponoharpalusdiscrepans*; **Ae**. Harpalus (Zangoharpalus) tinctulus luteicornoides; **Af**. *Saperdaoctomaculata*; **Ba**. *Acaloleptasejunctasejuncta*; **Bb**. *Protaetialugubris*; **Bc**. Paridea (Paridea) angulicollis; **Bd**. *Meligethesflavicollis*; **Be**. *Bruchidiusjaponicus*; **Bf.** *Demotinamodesta*; **Ca**. *Alticaoleraceaoleracea*; **Cb**. *Synetaadamsi*; **Cc.** *Epilachnaquadricollis*; **Cd.** *Orchestes* sp.; **Ce.** *Pseudocneorhinus* sp.; **Cf.** *Bradybatus* sp.; **Da.** Leiodidae sp.; **Db.** *Catops* sp.; **Dc.** *Sophropsstriata*; **Dd.** *Omositadiscoidea*; **De.** Epuraea (Epuraea) oblonga; **Df.** *Neopallodesomogonis*; **Ea.** Glischrochilus (Librodor) rufiventris; **Eb.** Ipidia (Ipidia) variolosa variolosa; **Ec.** Aspidobyctiscus (Aspidobyctiscus) lacunipennis; **Ed.** *Blitoperthaorientalis*; **Ee.** *Salpingusdepressifrons*; **Ef.** *Uleiotaarboreus*; **Fa.** Allecula (Upinella) melanaria; **Fb.** *Lagrianigricollis*; **Fc.** Mycetochara (Ernocharis) orientalis; **Fd.** *Lagriarufipennis*; **Fe.** *Euborelliaannulata*; **Ff.** Agromyzidae sp.; **Ga.** *Bibio* sp.1; **Gb.** *Bibio* sp.2; **Gc.** *Bibiotenebrosus*; **Gd.** Cecidomyiidae sp.; **Ge.** Chloropidae sp; **Gf.** *Coelopafrigida*; **Ha.** *Condylostylusnebulosus*; **Hb.** Dolichopodinae sp.; **Hc.** Ephydridae sp.; **Hd.** *Suilliabrunneipennis*; **He.** *Suillialineitergum*; **Hf.** Heleomyzidae sp.; **Ia.** *Suillia* sp.; **Ib.** *Homoneurafiliola*; **Ic.** *Homoneurahaejuana*; **Id.** *Sciasmomyiasupraorientalis*; **Ie.** *Homoneura* sp.; **If.** *Lonchoptera* sp.; **Ja.** *Dichaetomyiabibax*; **Jb.** *Atherigona* sp.; **Jc.** Mycetophilidae sp.; **Jd.** Phoridae sp.1; **Je.** Phoridae sp.2; **Jf.** *Euprosopiagraham*; **Ka.** *Psila* sp.; **Kb.** *Scathophagamellipes*; **Kc.** Sciaridae sp.; **Kd.** Sphaeroceridae sp. 1; **Ke.** Sphaeroceridae sp. 2; **Kf.** *Allognostavagans*; **La.** *Acanthonevratrigona*; **Lb.** *Alyduscalcaratus*; **Lc.** *Paraplesiusunicolor*; **Ld.** *Drabescusnigrifemoratus*; **Le.** *Drabescusnitobei*; **Lf.** Charagochilus (Charagochilus) angusticollis; **Ma.** *Bryocorismontanus*; **Mb.** *Castanopsides* sp.; **Mc.** Nabis (Milu) apicalis; **Md.** *Gardenabrevicollis*; **Me.** *Physatocheilafieberi*; **Mf.** *Technomyrmexgibbosus*; **Na.** *Lasiushayashi*; **Nb.** *Camponotuskiusiuensis*; **Nc.** *Formicalemani*; **Nd.** *Stenammaowstoni*; **Ne.** *Cryptoponesauteri*; **Nf.** *Temnothoraxspinosior*; **Oa.** *Temnothorax* sp.; **Pa.** Coelichneumon (Coelichneumon) cyaniventris; **Pb.** *Pataniachlorophanta*; **Pc.** *Manuleajaponica*; **Pd.** *Thyasjuno*; **Pe.** *Catocalalara*; **Pf.** *Miltochristaminiata*; **Qa**. *Hypocalasubsatura*; **Qb.** Erebidae sp.; **Qc.** *Problepsisdiscophora*; **Qd.** *Pachyligiadolosa*; **Qe.** *Bistonrobustum*; **Qf.** *Orthosiaaskoldensis*; **Ra.** *Diarsiadeparca*; **Rb.** *Dictyestradissecta*; **Rc.** *Euplexialucipara*; **Rd.** *Dimorphicosmiavariegate*; **Re.** *Amyna* sp.; **Rf.** Teleogryllus (Brachyteleogryllus) emma. **Scale bars**: Aa-Af, Bc-Mc, Me-Oa = 1.0 mm; Ba, Bb, Md, Pa-Rf = 1.0 cm.

This survey identified 10 orders, 105 families, 216 genera and 212 species of insects (Table 1, Fig. 2). This list includes 12 families, two subfamilies, 13 genera and 74 species that have not been previously recorded on Ulleungdo.

The largest number of unrecorded species belonged to Coleoptera (28 species), followed by Lepidoptera (14 species), Diptera (13 species), Hemiptera (9 species) and Hymenoptera (8 species). Additionally, one previously unrecorded species each of Dermaptera and Orthoptera were found. In Diptera, 11 families, one subfamily and seven genera that have not been classified to the species level were identified. If all of these were to be identified at the species level, at least 32 unrecorded species would be recorded. Furthermore, Diptera appears to be the taxon with the highest possibility of unrecorded species being discovered. In the Braconidae family of Hymenoptera, Pyralidae family of Lepidoptera and Trichoptera, identification to the species level was difficult owing to the lack of experts. If accurate identification could be achieved, further previously unrecorded species would be identified.

The unrecorded species identified in this survey include pests, such as *Aspidobyctiscuslacunipennis* and *Euplexialucipara*, which infest crops, such as grapes and beans and *Bistonrobustum*, *Pachyligiadolosa* and *Pataniachlorophanta*, which infest forests, such as oak, camellia and persimmon (Lim et al. 2013, National Institute of Biological Resources 2022, Korea Forest Service 2022). The results of this survey highlight the necessity of obtaining the latest insect fauna data in Ulleungdo, updating the insect fauna through continuous monitoring, preventing the introduction of pests and implementing efforts to minimise damage to crops and forest resources.

### Conflicts of interest

The authors have no conflicts of interest to declare.

## Figures and Tables

**Table 1.** Ulleungdo insect list in 2020.

**Order**	**Family**	**Subfamily**	**Scientific name**	**Newly-recorded**			
				**Family**	**Subfamily**	**Genus**	**Species**
Blattodea							
	Ectobiidae						
			*Blattellanipponica*				
Coleoptera							
	Anthribidae						
			Anthribidae sp.				
	Aphodiidae						
			*Saprositesjaponicus*				O
	Bostrichidae						
			Bostrichidae sp.	O			
	Brentidae						
		Apioninae					
			Apioninae sp.		O		
	Buprestidae						
			*Agrilus* sp.				
			*Agriluschujoi*				
	Carabidae						
			*Parena* sp.				
			*Synuchus* sp.				
			Anisodactylus (Pseudanisodactylus) signatus				
			Amara (Curtonotus) giganteus				
			Amara (Amara) ussuriensis				
			*Gyrochaetostylusatricomes*				O
			Harpalus (Harpalus) chalcentus				
			Harpalus (Zangoharpalus) tinctulus luteicornoides				O
			Lesticus (Triplogenius) magnus				
			*Metacolpodesbuchannani*				
			*Nipponoharpalusdiscrepans*				O
	Cerambycidae						
			*Acaloleptasejunctasejuncta*				O
			Anaglyptus (Aglaophis) colobotheoides				
			*Arhopaloscelisbifasciata*				
			*Arhopalusrusticusrusticus*				
			Egesina (Niigimaia) bifasciana bifasciana				
			*Mimectatinadivaricatadivaricata*				
			*Saperdaoctomaculata*				O
	Scarabaeidae						
			*Blitoperthaorientalis*				O
			*Protaetialugubris*				O
			*Sericania* sp.				
			*Sericaniafuscolineata*				
			*Sophropsstriata*				O
	Chrysomelidae						
			Chrysomelidae sp.				
			*Cryptocephalus* sp.				
			*Alticaoleraceaoleracea*				O
			*Argopistestsekooni*				
			*Bruchidiusjaponicus*				O
			*Demotinamodesta*				O
			*Gallerucidabifasciata*				
			*Pagriasignata*				
			Paridea (Paridea) angulicollis				O
			*Synetaadamsi*				O
	Coccinellidae						
			*Calviamuiri*				
			*Epilachnaquadricollis*				O
			*Harmoniaaxyridis*				
			Illeis (Illeis) koebelei koebelei				
	Curculionidae						
			Curculionidae sp.			O	
			*Bradybatus* sp.				
			*Orchestes* sp.			O	
		Entiminae					
			Entiminae sp.				
			*Pseudocneorhinus* sp.			O	
			*Pseudoedophryshilleri*				
	Elateridae						
			Elateridae sp.				
			*Melanotus* sp.				
			*Drasteriusagnatus*				
			*Pectocerafortunei*				
	Endomychidae						
			*Ancylopuspictusasiaticus*				
	Hydrophilidae						
		Spaeridiinae					
			Spaeridiinae sp.				
	Leiodidae						
			Leiodidae sp.			O	
			*Catops* sp.	O			
	Lucanidae						
			*Dorcusrectusrectus*				
	Meloidae						
			Meloidae sp.				
			Meloe (Meloe) proscarabaeus proscarabaeus				
	Mordellidae						
			Mordellidae sp.				
	Nitidulidae						
			Nitidulidae sp.				
			Epuraea (Epuraea) oblonga				O
			Glischrochilus (Librodor) rufiventris				O
			Ipidia (Ipidia) variolosa variolosa				O
			*Meligethesflavicollis*				O
			*Neopallodesomogonis*				O
			*Omositadiscoidea*				O
	Rhynchitidae						
			Aspidobyctiscus (Aspidobyctiscus) lacunipennis				O
	Salpingidae						
			*Salpingusdepressifrons*				O
	Scraptiidae						
			Scraptiidae sp.				
	Silphidae						
			Necrophila (Eusilpha) jakowlewi jakowlewi				
	Silvanidae						
			*Uleiotaarboreus*				O
	Staphylinidae						
			Staphylinidae sp.				
			Aleochara (Aleochara) curtula				
		Tachyporinae					
			Tachyporinae sp.				
	Tenebrionidae						
			Allecula (Upinella) melanaria				O
			Gonocephalum (Gonocephalum) pubens				
			Lagria (Lagria) nigricollis				O
			Lagria (Lagria) rufipennis				O
			*Lupropsorientalis*				
			Mycetochara (Ernocharis) orientalis				O
Dermaptera							
	Anisolabididae						
			*Euborelliaannulata*				O
	Forficulidae						
			*Anechurajaponica*				
	Anisolabididae						
			*Anisolabellamarginalis*				
Diptera							
	Agromyzidae						
			Agromyzidae sp.	O			
	Anisopodidae						
			*Sylvicolajaponicus*				
	Anthomyiidae						
			Anthomyiidae sp.				
			*Deliaplatura*				
			*Fucelliaapicalis*				
	Asilidae						
		Ommatiinae					
			Ommatiinae sp.				
	Bibionidae						
			*Bibio* sp.1			O	
			*Bibio* sp.2			O	
			*Bibiotenebrosus*				O
	Calliphoridae						
			Calliphoridae sp.				
			*Lucilia* sp.				
	Cecidomyiidae						
			Cecidomyiidae sp.	O			
	Chloropidae						
			Chloropidae sp.	O			
	Coelopidae						
			*Coelopafrigida*				O
	Dolichopodidae						
			*Condylostylusnebulosus*				O
		Dolichopodinae					
			Dolichopodinae sp.		O		
	Drosophilidae						
			Drosophilidae sp.				
			*Drosophila* sp.				
			*Scaptomyza* sp.				
	Ephydridae						
			Ephydridae sp.	O			
	Fanniidae						
			*Fannia* sp.				
	Heleomyzidae						
			Heleomyzidae sp.	O			
			*Suillia* sp.			O	
			*Suilliabrunneipennis*				O
			*Suillialineitergum*				O
			*Suillianartshukella*				
	Lauxaniidae						
			Lauxaniidae sp.				
			*Homoneura* sp.			O	
			*Homoneurafiliola*				O
			*Homoneurahaejuana*				O
			*Sciasmomyiasupraorientalis*				O
	Lonchopteridae						
			*Lonchoptera* sp.			O	
	Muscidae						
			*Atherigona* sp.			O	
		Muscinae					
			Muscinae sp.				
		Coenosiinae					
			*Lispe* sp.				
		Phaoniinae					
			Phaoniinae sp.				
			*Dichaetomyiabibax*				O
	Mycetophilidae						
			Mycetophilidae sp.	O			
	Phoridae						
			Phoridae sp.1	O			
			Phoridae sp.2	O			
	Platystomatidae						
			*Euprosopiagrahami*				O
			*Rivelliaalini*				
			*Rivellianigroapicalis*				
	Psilidae						
			*Psila* sp.			O	
	Psychodidae						
			Psychodidae sp.				
	Sarcophagidae						
			Sarcophagidae sp.				
		Sarcophaginae					
			Sarcophaginae sp.				
	Scatophagidae						
			*Scathophaga* sp.				
			*Scathophagamellipes*				O
			*Scathophaga Stercoraria*				
	Sciaridae						
			Sciaridae sp.	O			
	Simuliidae						
			*Simulium* sp.				
	Sphaeroceridae						
			Sphaeroceridae sp 1.	O			
			Sphaeroceridae sp 2.	O			
	Stratiomyidae						
			*Allognostavagans*				O
	Syrphidae						
			Syrphidae sp.				
	Tachinidae						
			Tachinidae sp.				
			*Tachina* sp.				
	Tephritidae						
			Tephritidae sp.				
			*Campiglossa* sp.				
			*Acanthonevratrigona*				O
			*Anomoiapurmunda*				
Hemiptera							
	Acanthosomatidae						
			Acanthosomatidae sp.				
			*Acanthosomacrassicaudum*				
			*Acanthosomadenticaudum*				
			*Acanthosomaforficula*				
			*Elasmostethusnubilus*				
			*Sastragalascutellata*				
	Achilidae						
			*Erradanawae*				
	Alydidae						
			*Alyduscalcaratus*				O
			*Paraplesiusunicolor*				O
			*Riptortusclavatus*				
	Aphididae						
			Aphididae sp.				
	Aphrophoridae						
			*Obiphoraintermedia*				
	Cicadellidae						
			Cicadellidae sp.				
			*Cicadellaviridis*				
			*Drabescusnigrifemoratus*				O
			*Drabescusnitobei*				O
			Idiocerus (Bicenarus) ishiyamae				
			*Neotituriakongosana*				
			*Phlogotettixcyclops*				
	Cicadidae						
			*Meimunaopalifera*				
	Coreidae						
			Homoeocerus (Tliponius) dilatatus				
	Cydnidae						
			*Macroscytusjaponensis*				
	Delphacidae						
			Delphacidae sp.				
			*Stenocranus* sp.				
			*Sogatellafurcifera*				
	Lygaeidae						
			Lygaeidae sp.				
			*Neolethaeusdallasi*				
			*Nysiusplebejus*				
	Miridae						
			Miridae sp.				
			*Castanopsides* sp.			O	
			Charagochilus (Charagochilus) angusticollis				O
			*Bryocorismontanus*				O
			*Monalocorisfilicis*				
	Nabidae						
			Nabis (Milu) apicalis				O
			Nabis (Nabis) stenoferus				
	Pentatomidae						
			*Aeliafieberi*				
			*Aeliaklugii*				
			*Glauciassubpunctatus*				
			*Leliadecempunctata*				
			*Menidascotti*				
			*Plautiastali*				
			*Zicronacaerulea*				
	Psyllidae						
			Psyllidae sp.				
	Reduviidae						
			Reduviidae sp.				
			*Gardenabrevicollis*				O
	Ricaniidae						
			*Orosangajaponica*				
	Tingidae						
			*Physatocheilafieberi*				O
Hymenoptera							
	Andrenidae						
			Andrenidae sp.				
	Apidae						
			Apidae sp.				
			*Apismellifera*				
			Bombus (Pyrobombus) ardens ardens				
			*Bombusspeciosus*				
	Braconidae						
			Braconidae sp.				
	Formicidae						
			*Camponotus* sp.				
			*Camponotusitoi*				
			*Camponotusjaponicus*				
			*Camponotuskiusiuensis*				O
			*Formicalemani*				O
			*Lasiusalienus*				
			*Lasiushayashi*				O
			*Lasiusspathepus*				
			*Nylanderiaflavipes*				
			*Stigmatommasilvestrii*				
			*Technomyrmexgibbosus*				O
		Myrmicinae					
			Myrmicinae sp.				
			*Temnothorax* sp.			O	
			*Pheidolefervida*				
			*Pristomyrmexpunctatus*				
			*Stenammaowstoni*				O
			*Temnothoraxspinosior*				O
			*Tetramoriumtsushimae*				
		Ponerinae					
			Ponerinae sp.				
			*Cryptoponesauteri*				O
	Ichneumonidae						
			Ichneumonidae sp.				
			Coelichneumon (Coelichneumon) cyaniventris				O
	Vespidae						
			*Vespasimillimasimillima*				
			*Vespulaflavicepsflaviceps*				
Lepidoptera							
	Callidulidae						
			*Pterodectafelderi*				
	Crambidae						
			*Glyphodespryeri*				
			*Glyphodesquadrimaculalis*				
			*Haritalodesderogata*				
			*Herpetogrammaluctuosalis*				
			*Paligaauratalis*				
			*Palpitanigropunctalis*				
			*Pataniachlorophanta*				O
	Drepanidae						
			*Nordstromiajaponica*				
			*Thyatirabatisbatis*				
	Erebidae						
			Erebidae sp.	O			
			*Barsinestriata*				
			*Catocalalara*				O
			*Catocalanubila*				
			*Chionarctianivea*				
			*Hypenaamica*				
			*Hypocalasubsatura*				O
			*Manuleajaponica*				O
			*Miltochristaminiata*				O
			*Spilarctiaseriatopunctata*				
			*Thyasjuno*				O
	Geometridae						
			Geometridae sp.				
			*Abraxasfulvobasalis*				
			*Bistonrobustum*				O
			*Caberagriseolimbata*				
			*Deilepteniaribeata*				
			*Dysstromajaponica*				
			*Epirrhoesupergressa*				
			*Gandaritisfixseni*				
			*Lobogonodeserectaria*				
			*Lomographabimaculata*				
			*Lomographatemerata*				
			*Odontoperaarida*				
			*Orthocaberatinagmaria*				
			*Ourapteryxkoreana*				
			*Pachyligiadolosa*				O
			*Phthonosematendinosaria*				
			*Problepsisdiscophora*				O
	Lycaenidae						
			*Cupidoargiades*				
			*Pseudozizeeriamaha*				
	Noctuidae						
			Noctuidae sp.				
			Amyna sp.			O	
			*Amphipyralivida*				
			*Antoculeoralocuples*				
			*Athetislineosa*				
			*Callopistriarepleta*				
			*Chasminodesalbonitens*				
			*Chrysodeixiseriosoma*				
			*Ctenoplusiaalbostriata*				
			*Diarsiacanescens*				
			*Diarsiadeparca*				O
			*Dictyestradissecta*				O
			*Dimorphicosmiavariegata*				O
			*Dypterygiacaliginosa*				
			*Euplexialucipara*				O
			*Orthosiaaskoldensis*				O
			*Orthosiacarnipennis*				
			*Sineugrapheoceanica*				
			*Xestiac-nigrum*				
			*Xestiaefflorescens*				
	Notodontidae						
			*Epodontalineata*				
			*Euhampsoniacristata*				
			*Spataliaplusiotis*				
	Nymphalidae						
			*Kaniskacanace*				
			*Minoisdryas*				
	Pieridae						
			*Anthocharisscolymus*				
			*Pierisrapae*				
	Pyralidae						
			Pyralidae sp.				
	Saturniidae						
			*Samiacynthia*				
	Sphingidae						
			*Acosmeryxnaga*				
			*Ambulyxjaponicakoreana*				
			*Callambulyxtatarinovii*				
	Tortricidae						
			Tortricidae sp.				
Mantodea							
	Mantidae						
			*Tenoderasinensis*				
							
Orthoptera							
	Acrididae						
			*Shirakiacrisshirakii*				
			*Trilophidiaannulata*				
	Gryllidae						
			Gryllidae sp.				
			*Oecanthuslongicauda*				
			Teleogryllus (Brachyteleogryllus) emma				O
	Rhaphidophoridae						
			Paratachycines (Paratachycines) ussuriensis				
			Tachycines (Tachycines) coreanus				
	Tetrigidae						
			*Tetrixjaponica*				
	Tettigoniidae						
			Tettigoniidae sp.				
			*Ducetiajaponica*				
			*Hexacentrusjaponicus*				
			*Phaneropterafalcata*				
			*Phaneropteranigroantennata*				
Trichoptera							
			Trichoptera sp.				
